# Advances in the Mechanistic Study of the Control of Oxidative Stress Injury by Modulating HDAC6 Activity

**DOI:** 10.1007/s12013-022-01125-w

**Published:** 2023-02-07

**Authors:** Yuanye Xue, Bing Gan, Yanxing Zhou, Tingyu Wang, Tong Zhu, Xinsheng Peng, Xiangning Zhang, Yanfang Zhou

**Affiliations:** 1 Department of Pathophysiology, Guangdong Medical University, Dongguan, 523808 China; 2 The Third Affiliated Hospital of Guangdong Medical University, Fo Shan, 528000 Guangdong China; 3 School of Medical Technology, Guangdong Medical University, Dongguan, 523808 China; 4 Biomedical Innovation Center, Guangdong Medical University, Dongguan, 523808 China; 5 Institute of Marine Medicine, Guangdong Medical University, Zhanjiang, 524023 China

**Keywords:** Protein modification, Acetylation, Histone deacetylase 6 (HDAC6), Oxidative stress, Signaling pathway

## Abstract

Oxidative stress is defined as an injury resulting from a disturbance in the dynamic equilibrium of the redox environment due to the overproduction of active/radical oxygen exceeding the antioxidative ability of the body. This is a key step in the development of various diseases. Oxidative stress is modulated by different factors and events, including the modification of histones, which are the cores of nucleosomes. Histone modification includes acetylation and deacetylation of certain amino acid residues; this process is catalyzed by different enzymes. Histone deacetylase 6 (HDAC6) is a unique deacetylating protease that also catalyzes the deacetylation of different nonhistone substrates to regulate various physiologic processes. The intimate relationship between HDAC6 and oxidative stress has been demonstrated by different studies. The present paper aims to summarize the data obtained from a mechanistic study of HDAC6 and oxidative stress to guide further investigations on mechanistic characterization and drug development.

## Introduction

Oxidative stress is defined as a disequilibrium in active oxygen levels and antioxidative defense [1]. Reactive oxygen species (ROS) are generated during the normal oxidation of cell metabolism; these factors include singlet oxygen (1O_2_), superoxide anion, hydroxyl free radicals (·OH) and hydrogen peroxide (H_2_O_2_) [2]. Under physiological conditions, the level of ROS in mammalian cells is tightly regulated because the antioxidative system can eliminate high levels of ROS. Reduced antioxidative ability and increased production of active oxygen, however, may result from environmental pressure, including inflammation, hypoxia/ischemia, the release of active neurotransmitters or mitochondrial damage [3–5]. If uncontrolled, the accumulated active oxygen disturbs redox homeostasis [6] to induce oxidative stress and oxygenate nucleic acids, lipids and proteins that are essential for normal cell functions, leading to organelle injury and tissue dysfunction [7, 8].

ROS mediate multiple signaling pathways, including progression of the cell cycle, inflammation, MAPK signaling and apoptosis [9–12]. Increased ROS production is implicated in the development of different diseases, including cardiovascular disorders [13], mitochondrial cristae injury [14], inflammation [15], joint degeneration [16], ischemia‒reperfusion injury [17], renal injury [18], diabetes mellitus (DM) [19], and damage to the retina and spine [20, 21]. A reduction in the generation of active oxygen, therefore, significantly contributes to controlling the development of diseases.

Histone deacetylases (HDACs) are a group of enzymes that remove acetyl groups from protein molecules to regulate various biological functions [22]. HDAC6 is a class II HDAC with cytoplasmic localization and two catalytic domains [23]. It has been demonstrated that HDAC6 plays an important role in the regulation of redox activity and oxidative stress [24]. Elevated HDAC6 activity due to mitochondrial dysfunction [17, 25] and alterations in endogenous antioxidative function [24] cause oxidative stress. The active role of gene knockout and inhibitors of HDAC6 on regulation of oxidative stress has also been verified [6, 25, 26]. Inhibition of HDAC6, however, stimulates oxidative stress in some cells [27]. Further investigations are needed on the modulation of oxidative stress mediated by HDAC6 and the underlying mechanisms. The present paper aims to review the pathways by which HDAC6 regulates oxidative stress and alterations in relevant proteins.

## Acetylation and HDAC6

The dynamic equilibrium of acetylation and deacetylation plays a crucial role in the maintenance of normal physiologic functions; this is an abundant and important posttranslational modification that acts as the regulatory signal in many cellular processes [28]. Protein acetylation mediates a variety of functions, including protein‒protein interactions, such as transcription initiation and control, tumor suppression, subcellular localization and transcription potential [29–31]. The acetylation levels of histone and nonhistone proteins are modulated by HDACs and histone acetyltransferases (HATs). The modification is completed by transferring the acetyl group from acetyl-coenzyme A to the ε-amino group on the lysine residue of the polypeptide chain and is catalyzed by HATs. The activity of HATs is antagonized by HDACs; these two types of enzymes dynamically maintain the equilibrium of acetylation in the body [30]. HDACs contribute to epigenetic and signal modification by catalyzing the removal of acetyl groups from amino acid residues [32], which is closely related to cell growth, dedifferentiation, proliferation and apoptosis [33, 34]. Mammalian HDACs are divided into Classes I, II, III, and IV according to their homology with yeast HDACs. Classes I, II, and IV HDACs are Zn^2+-^dependent with distinctive structures that are nicotinamide adenine dinucleotide (NAD^+^) dependent. Class III HDACs are called sirtuins [35].

HDAC6 belongs to the Class II family of HDAC, which is composed of 18 members; it contains two homologous catalytic domains and a C-terminal ubiquitin binding zinc finger domain [36, 37]. HDAC6 mainly deacetylates nonhistone proteins. Known HDAC6 substrates include α-tubulin [37], cortactin [38], peroxiredoxin [24, 35])] and heat shock protein 90 (HSP90) [39]], together with membrane proteins such as EGFR [40] and GRK2 [41]. HDAC6 may also migrate to nucleus to bind to survivin [42].

HDAC6 is a stress monitoring factor that affects multiple cellular events, including the degradation of incorrectly folded proteins, DNA repair, cell adhesion, cell expansion and migration, the heat shock response, inflammation, autophagy, microtubule-dependent transport, pressure particle formation and the formation of immune synapses [11, 24, 43, 44, 45, 46, 47, 48, 49]. HDAC6 also participates in the innate immune response, including antiviral immunity and macrophage activation [50, 51]), the mediation of immunity triggered by different microbe products, such as Clostridium difficile toxin A, lipopolysaccharide (LPS) and HIV-1 Tat [43, 52, 53, 54], and acts as an important regulator of antibacterial and antiviral immune responses. In addition, HDAC6 impacts cellular redox status by regulating the deacetylation levels of endogenous antioxidants and oxidase activity [17]. Disturbances in HDAC6 lead to the occurrence of various diseases, such as inflammation [48], nerve degeneration [55], cardiovascular disorders [28] and cancer [56, 57]. Overexpression of HDAC6 is correlated with the pathological grading and staging of tumors [3], as well as metastatic potential [18,58]. HDAC6 inhibition is a promising therapeutic approach for many diseases and is used to treat diseases such as neural degenerative disorders [20, 25], ciliary and heart dysfunction [59, 30], dementia [60], DM [61], lung injury [8], chronic kidney disease [62], spinal injury [53] and cancer [56].

## The Pathways that Reduce Oxidative Stress by Regulating HDAC6

### HDAC6 and prx

Peroxiredoxin (Prx) maintains intracellular redox homeostasis to protect the cell against injury due to radical accumulation; it catalyzes the reduction of peroxidases to protect the cell from oxidative stress [63, 64]. The Prx family contains 6 antioxidant proteins: Prx 1-6 [65]. Prx possesses multiple functions. In addition to being involved in the regulation of processes including cell growth, metabolism, death and survival [66], it acts as a sensor of redox reactions; Prx peroxidation and inactivation may result from oxidative stress to reduce the cellular antioxidative potential [67].

Available data suggest that HDAC6 modulates the activity of Prx through acetylation to regulate oxidative injury.

Pretreatment with the HDAC6 inhibitor tubastatin A induces Prx1 activity in a dose-dependent manner in H_2_O_2-_challenged 661 W cells to ameliorate H_2_O_2-_mediated injury [66]. In Parkinson syndrome model mice induced by 6-OHDA, pharmacologic inhibition by tubastatin A induces the acetylation of Prx1 and Prx2 in dopaminergic neurons and decreases the production of ROS to protect dopaminergic neurons from 6-OHDA-induced death [68].

Further study suggests that Prx1-mediated reduction of peroxidase is dependent on acetylation mediated by HDAC6 [24, 63]. In primary hippocampal cells and HT22 cells, the acetylation of Prx1 is reduced in an Alzheimer’s disease (AD) model and restored by tubastatin A. The change in ROS correlates with the acetylation of Prx1 but is independent of the amount of Prx1. The data suggest that an increase in the level of acetylation shows more potential antioxidative and reduction activity. Further investigation showed that HT22 cells transfected with the mimic mutant K197Q partially resisted the increase in ROS in the AD model, while the acetylation silencing mutant exhibited similar results as the wild-type (WT) group.

In HT22 cells transfected with the silencing mutant of Prx1, coincubation with tubastatin A and Aβ did not restore the levels of ROS, but in wild-type Prx1. These findings suggest that inhibiting HDAC6 reduces the level of ROS by deacetylating Prx1 at K197 [69]. In addition, the change in Ca^2+^ is identical to the change in ROS, and the Ca^2+^ inhibitor BAPTA-AM reduces Aβ-mediated ROS increases, suggesting that the reduction in ROS caused by Prx1 acetylation is partially attributed to the reduction in Ca^2+^. Similar findings were obtained from in vivo experiments. In comparison with wild-type controls, an AD model in 5xFAD mice showed reduced Prx1 acetylation in brain tissues, but injection of an HDAC6 inhibitor reversed this reduction and reduced oxidative stress [69]. Similar observations have been recorded in ischemia‒reperfusion rats and cardiomyocytes [70].

### HDAC6 and Mitochondria

In most mammalian cells, mitochondria supply energy using ATP generated by phosphorylation-oxidation, which uses O_2_ as the ultimate electron receptor [71]. ROS are produced during this process [61]. The main ROS generated by mitochondria are O^2−^. A majority of O^2−^ is degraded to form H_2_O_2_ [72]. Research suggests that ROS production can be used to evaluate mitochondrial function [73].

Under normal conditions, ROS are maintained at normal levels through antioxidative system in cells. Excessive ROS production induces oxidative stress and oxygenates mitochondrial membrane proteins, altering the permeability of the outer membrane. Disturbances in mitochondrial membrane potential leads to the release cytochrome c, which triggers apoptosis [74]. The overproduction of ROS causes mitochondrial dysfunction and oxidative damage, further increasing the production of ROS. This detrimental feed-forward loop promotes disease progression [75]. HDAC6 activity contributes to mitochondrial dysfunction and oxidative stress [17, 25].

HDAC6 is known to modulate mitochondrial activity in many types of cells. For example, HDAC6 may cause the loss of the ability to eliminate damaged mitochondria by autophagy [76]. In RAW264.7 cells that were pretreated with the HDAC6 inhibitor ACY-1215, changes in mitochondrial ultrastructure and the mitochondrial membrane induced by lipopolysaccharide (LPS) were restored to normal [77].

Evidence suggests that HDAC6 controls oxidative stress injury by modulating mitochondria. Superoxides and endogenous thioredoxin 1 (Trx1) derived from mitochondria are elevated in HuREC cells (human retinal endothelial cells) cultured in high glucose medium, and these negative effects were inhibited by tubastatin A [20]. Proteomics data reveal a marked decrease in the mitochondrial inner membrane and protein complexes in an acute liver failure (ALF) mouse model, while treatment with the HDAC6 inhibitor ACY-1215 reversed these changes, reducing the production of ROS in ALF mice and reducing liver cell necrosis; the effect was achieved by normalizing the activity of mitochondrial electron transmission chain complex I and restoring oxidation-phosphorylation in ALF mice [78].

PHB1 (prohibitin 1) is essential for proper mitochondrial function. Data indicate that in pyrotoxicemic patient-derived peripheral blood mononuclear cells (PBMCs) and pyrotoxicemic rats, PHB1 expression was negatively correlated with HDAC6 levels, and inhibiting HDAC6 ameliorated injury due to mitochondrial damage and pyrotoxicemia. It is thought that inhibiting mitochondria with PHB1 ameliorates the injury caused by pyrotoxicemia through HDAC6 regulation [79].

Another study revealed that HDAC6 modulates mitochondrial function through ROS. In A375.S2 cells, HDAC6 inhibition by siRNA resulted increased ROS levels, decreased mitochondrial membrane potential, reduced the level of PGC-1, the key regulatory element in mitochondrial biogenesis, increased the mitochondrial fusion protein Mfn2 and reduced the mitochondrial division protein DRP1. These changes were restored by an ROS inhibitor. Mitochondrial fusion and division are key processes associated with the continuous remodeling of the mitochondrial network, suggesting that HDAC6 modulates mitochondrial function through ROS [25].

Contradictory results have been obtained from different studies. Evidence directly suggests that in human melanoma cells that highly express HDAC6, knockdown triggers marked ROS production and a decrease in mitochondrial membrane potential [25]. In A549 cells, knockdown of HDAC6 reduces mitochondrial enzymatic activities [80]. Another study suggests that in mesenchymal stem cells (MSCs), knockdown of HDAC6 elevates the level of ROS. This finding may be explained by disturbances in the oxidative metabolism of mitochondria [81]. HDAC6-knockout mice exhibit no phenotype related to mitochondrial defects [76, 82, 83]. This finding indicates that HDAC6 maintains intact mitochondria during stress conditions [84].

### HDAC6 and Autophagy

Autophagy is a lysosome-dependent process that degrades metabolites, and dysfunctional organelles, misfolded proteins and radical oxygen are reused to maintain cellular homeostasis [85]. Autophagy is an efficient adaptation process that protects cells from injury caused by various stimuli, including lysosomes, the degradation of dysfunctional organelles and protein clusters, and oxidative stress [44, 86]. When organelles and proteins are engulfed in the autophagosome, they fuse with lysosomes, and the target substances are hydrolyzed by enzymes within lysosomes [45, 87]. Moreover, autophagy is a key cellular event that antagonizes mitochondrial damage mediated by oxidative stress [88]. In mammalian cells, the accumulation of mitochondrial ROS and lipid oxidation plays essential roles in autophagy, as both O2− and H2O2 trigger autophagy [46]. Autophagy plays an important role in maintaining normal oxidation and reduction equilibrium through the degradation of substances produced by injured cells [44, 86].

The link between HDAC6 and autophagy has been demonstrated, but different results have been presented in different studies. Some data suggest that inhibiting HDAC6 reduces autophagy. By recruiting epidermal actin-dependent actin‑remodeling machinery, recombinant actin stimulates the fusion of lysosomes and autophagosomes through the assembly of an F-actin network to degrade substrates [89, 90]. For example, inhibiting HDAC6 weakens autophagy induced by bortezomib in HNSCC cells [91], the HDAC6 inhibitor tubacin induces autophagy and blocks the fusion of autophagosomes [92, 93]. Autophagy is classified as macroautophagy, microautophagy, and chaperone-mediated autophagy (CMA) according to how proteins are transported and whether a double membrane is formed. CMA is activated constitutively and maximally under oxidative stress [94], providing a defense against oxidative stress [95]. Knockout or knockdown of HDAC6 induces irreversible hyperacetylation of HSP90, leading to decreased CMA activity and weakening cellular resistance to ROS [96].

Some studies have suggested that HDAC6 inhibition increases autophagy. In an acute renal injury rat model, the number of autophagosomes formed by Atg7 and Beclin-1 was markedly elevated in injured kidneys. Treatment with tubastatin A further increased the expression of Atg7 and Beclin-1, indicating that autophagy due to renal injury was increased by HDAC6 inhibition [97]. In CAL27 cells treated with the HDAC6 inhibitor ricolinostat for 24 h, cytoplasmic autophagosomes and lysosomes increased significantly, suggesting that ricolinostat induced autophagy in CAL27 cells [27]]. Another study revealed that HDAC inhibition promoted autophagy through the activation of transcription factor EB (TFEB) [98]. Further investigation revealed that after treatment with hydrogen peroxide, rat chondrocyte antioxidases and apoptosis induced by tubastatin A were inhibited by the autophagy inhibitor 3-MA, demonstrating that HDAC6-mediated antioxidative activity involved autophagy [6].

### HDAC6 and Nrf2

The transcription factor nuclear factor red cell 2 related factor (Nrf2) is rich in leucine zippers and belongs to the CNC motif transcription factor family. Nrf2 is a central regulator of cellular stress [99] and is expressed in various tissues and cells to protect against cell injury, particularly oxidative stress and imbalances in oxidation and reduction [100]. Oxidative and pro-electron agents bind Keap1 (Kelch-like ECH-associated protein 1), giving rise to the accumulation and translocation of Nrf2 in the nucleus [101]. Nrf2 contains a basic leucine zipper (bZIP) at its C-terminus, which functions as a DNA binding domain. It dimerizes with Mafin in the nucleus to recognize and bind the sequence GCTGAGTCA on the antioxidant module ARE and initiate transcription and the upregulation of cytoprotective and antioxidative enzymes mediated by ARE, including cysteine S transferase (CST), catalase (CAT), thioredoxin (TRX), superoxidase (SOD), heme oxygenase-1 (HO-I), NAD(P)H, quinone oxidoreductase-1 (NQO1) and other antioxidases [55]. The expression of these factors helps to clear ROS, increase the synthesis of cysteine, and reduce ketones to protect cells from oxidative stress and maintain the equilibrium of intracellular partial oxygen in cells.

HDAC6 has been shown to inhibit the transcription factor Nrf2. In LPS-stimulated RAW264.7 cells, Nrf2 and the downstream protein HO-1 are restored to normal levels by inhibiting HDAC6 [77]. Nrf2 modulates the level of HDAC6, and HDAC6 is persistently upregulated in genetic oxidative-sensitive model mice with Nrf2 knockout [102].

Multiple investigations have revealed that HDAC6 modulates Nrf2 to ameliorate injury due to oxidative stress. After HDAC6 knockout, MCAO mice showed markedly elevated levels of Nrf2 and HO-1 in the cytosol and nucleus, suggesting that HDAC6 interference not only promotes the production of Nrf2 and accelerates its transport to the nucleus from the cytoplasm but also reduces the levels of oxidative stress markers in the serum, such as 3-NT, 4-HNE, and 8-OHdG, to protect mice from oxidative stress injury by activating the Nrf2 pathway in the context of HDAC6 inhibition [103]. Another study suggested that the levels of Nrf2-dependent genes during diabetes, such as HO-1, NQO1, glutamate-cysteine ligase regulatory subunit (GCLM) and glutamate-cysteine ligase (GCLC), as well as the oxidative markers DHE, NT, 4-HNE, and Trx1, are restored by tubastatin A, suggesting that tubastatin A reduces oxidative stress by restoring the Nrf2-dependent pathway in the diabetic retina [20].

### HDAC6 and NADPH Oxidase

NADPH oxidase is a potential source of intracellular ROS; it is composed of a membrane association subunit (gp91^phox^/Nox2, p22^phox^) and a cytoplasmic subunit (p40^phox^, p47^phox^, p67^pho^, small GTPase Rac) [104]. Upon activation, the cytoplasmic subunit is transferred to the membrane to bind gp91^phox^/Nox2 and then catalyzes the NADPH-dependent formation of superoxide from oxygen [105, 106].

Available data suggest that HDAC6 modulates the activity of NADPH oxidase to reduce oxidative stress-induced injury. In LPS-stimulated primary bovine mammary epithelial cells (bMECs), inhibiting HDAC6 with tubastatin A reduced the production of NADPH oxidase and the generation of ROS [107]. In RAW 264.7 cells, the overexpression of HDAC6 increased NADPH oxidase and ROS, and a high dose of an HDAC6 inhibitor plus NADPH oxidase reduced the production of ROS, directly indicating that the increase in ROS induced by HDAC6 is mediated in part by NADPH oxidase. In addition, the overexpression of HDAC6 activates the MAPK/NF-κB/AP-1 signaling pathway, and inhibitors of ROS and NADPH oxidase block the activation of MAPK, suggesting that HDAC6 overexpression influences the ROS-MAPK-NF-κB/AP-1 pathway to modulate inflammation by upregulating NADPH oxidase [11]. Further study revealed that knockout of HDAC6 and treatment with tubastatin A significantly inhibited HIV-1 Tat-induced NADPH oxidase activity and elevated ROS levels in CRT-MG human astroglial cells. Moreover, inhibitors of NADPH oxidase and ROS reduced the expression of HDAC6 in HIV-1 Tat-stimulated astrocytes, suggesting interactions between NADPH oxidase, ROS and HDAC6 [105].

### HDAC6 and XBP1

XBP1s (X box-binding protein-1) is a leucine zipper transcription factor that participates in mammalian UP and plays an important role in the antioxidative effect of the HDAC6 inhibitor tubastatin A. The accumulation of misfolded proteins due to endoplasmic reticulum (ER) errors triggers ER stress (ERS) and causes the unfolded protein response (UPR) [108]. ROS production is often related to transcriptional activation induced by ERS. Research indicates that tubastatin A and tubacin treatment increase the level of XBP1s and its acetylation in a dose- and time-dependent manner; the half-life of XBP1s is prolonged by approximately 30 min after tubastatin A treatment, and the expression of downstream genes significantly increases. The activity of XBP1s is elevated after challenge with H2O2 and tubastatin A, and inhibiting XBP1s with siRNA reduces the upregulation of antioxidative genes caused by tubastatin A, directly indicating the involvement of the antioxidant activity of HDAC6 [47, 100].

**Fig. 1:**
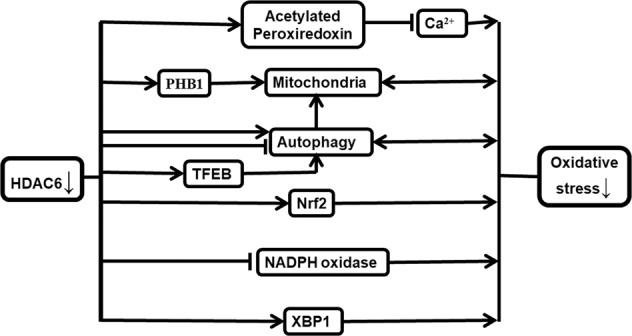
The deacetylase HDAC6 ameliorates oxidative stress through molecular interactions and biological process inhibition. The arrows denote the molecules and processes activated by HDAC6, while the vertical lines denote blocking or inhibition

The processes by which HDAC6 participates in ameliorating oxidative stress are depicted in Fig. 1.

### Other Oxidative Stress Pathways Related to HDAC6

In addition to the molecules and pathways discussed above, there are some molecules that indirectly contribute to HDAC6-mediated oxidative stress modulation. Their roles in these processes remain to be validated.

#### Heat shock proteins (HSPs)

Cells produce HSPs in response to stress [109]. HSPs are highly conserved proteins that serve as molecular chaperones and the first-line defense against stress. These proteins are induced by heat shock factor 1 (HSF1). In unstimulated cells, HSF1 is present in the inhibitory complex of HDAC6 and HSP90 [66]. During stress, HSF1 dissociates from the complex, is activated and migrates to the nucleus and binds to heat shock protein genes to initiate their expression [110].

HSPs are induced by oxidative stress [111]. Martindal and Holbrook [112]). HSP25 and HSP70 play important roles in protection against retinal degeneration induced by oxidative stress [60, 113]. HSP90 is a molecular chaperone protein that plays a key role in the defense against oxidative stress [60]. Increased HSP90 synthesis provides a more potent defense against ROS-induced injury, and its degradation is determined by acetylation [66].

HDAC6 is linked with the activation of HSP genes. Treatment of 661 W cells with tubastatin A upregulated HSP70 and HSP25 by activating HSF1. In addition, inhibiting HDAC6 stimulates the chaperone function of HSP90 through superacetylation [66]. In K-562 and HL-60 cells, the HDAC6 inhibitor tubacin increases the level of HSP90 acetylation; the ROS inhibitor NAC reduces HDAC6, HSF1, HSP27, HSP70 and HSP90 levels, suggesting that oxidative stress is an effective inducer of HDAC6 and HSPs [92].

When 661 W cells were incubated with KRIBB11, an inhibitor of HSF1, HSP70 and HSP25 induction was inhibited under oxidative stress conditions. The protective effect of tubastatin A against H2O2-induced cytotoxicity, however, was not weakened, suggesting that these HSPs do not act alone and are not sufficient to prevent oxidation-mediated injury, and the contribution of other factors is required [66].

#### NLRP3 inflammasome

NLRP3 is a member of the Nod-like receptor family, which senses pathogens and injury. It forms a cytoplasmic multiprotein complex with the apoptosis-related microparticle protein ASC and caspase-1 to modulate the activation of IL-1β and IL-18 [114]. Most confirmed NLRP3 activators also stimulate the production of ROS. Antioxidants have also been proven to inhibit the activation of NLRP3, indicating that redox signals participate in its activation [115]. A study indicated that inactivation of the NLRP3 inflammasome helps cells recover from oxidative stress, inflammation and apoptosis induced by high glucose [116, 117]. Treatment with the HDAC6 inhibitor Cay downregulates NLRP3 and oxidative stress, as well as inflammation indicators. The data suggest that Cay may inhibit oxidative stress, inflammation, and apoptosis by modulating the inflammatory pathway of NLRP3 [118]. Pharmacologic inhibition of HDAC6 with tubastatin A inhibits the activation of NLRP3-mediated ROS production by 6-OHDA in SH-SY5Y cells, a human neuroblastoma cell line, and a mouse nigrostriatal system and weakens the neurotoxicity of dopaminergic neurons [119].

#### NF-κB pathway

Nuclear factor-κB (NF-κB) is a transcription factor with multiple regulatory functions in cells [120]. Its activation is modulated by IκB kinase and the phosphorylation and degradation of IκBα, which are two components of the NF-κB family [48]. ROS produced by macrophages activate the NF-κB pathway and trigger inflammation to upregulate proinflammatory factors, chemotactic factors, adhesion molecules and NADPH [11, 121].

The promotion of inflammation and oxidative stress by HDAC6 involves the NF-κB pathway. In macrophages, HDAC6 overexpression activates the NF-κB signaling pathway and the expression of proinflammatory cytokines [11]. In LPS-stimulated RAW264.7 cells, inhibiting HDAC6 restores MAPK and NFκB pathway activation [77]. Another study indicated that in high glucose-treated cells, treatment with the HDAC6 inhibitor Cay significantly reduced the nuclear expression of NF-κB p65 and p-IκB-α and increased cytoplasmic NF-κB p65 expression, with concurrent reductions in oxidative, inflammatory and apoptotic indicators, suggesting that HDAC6 inhibitors block oxidative stress, inflammation and apoptosis induced by high glucose by modulating the NF-κB pathway [118]. In LPS-stimulated primary cultured bovine mammary epithelial cells (bMECs), inhibiting HDAC6 with tubastatin A reduced the production of ROS and the phosphorylation levels of NF-κB p65 and IκB in a dose-dependent manner [107]. In an acute renal injury mouse model, NF-κB signaling was markedly increased in mouse kidneys and was accompanied by elevated Il-6 and TNF-α expression. Pharmacologic inhibition of HDAC6 by tubastatin A significantly reduced the abnormal phosphorylation and activation of NF-κB and the expression of inflammatory factors. The data indicate that after renal injury, tubastatin A, which is an inhibitor of HDAC6, reduces NF-κB phosphorylation and the expression of IL-6 and TNF-α to relieve renal inflammation [122].

#### MAPK-ERK pathway

The MAPK-ERK signaling pathway is a complicated network that plays an essential role in physiologic and pathologic activities and involves a series of cellular responses triggered by environmental and developmental signals, including cell survival, proliferation, differentiation, inflammation and apoptosis [58]. During inflammation, MAPK and other transcription factors are strictly regulated in an oxidation‒reduction-dependent manner [123].

It has been demonstrated that HDAC6 and the MAPK-ERK signaling pathway possess a close relationship. ERK1/2, which is the end effector of the ERK-MAPK pathway, interacts with HDAC6 in vivo [124]. ERK1/2 phosphorylates HDAC6 at serine 1035 [124], and HDAC6 deacetylates p38, which is then phosphorylated by ERK1/2 in response to proteasomal inhibition [125]. In addition, JNK1 maintains the expression of HDAC6 [126].

A study indicated that HDAC6 overexpression stimulated the MAPK signaling pathway, and the effect was blocked by ROS inhibitors, suggesting that HDAC6 overexpression modulates the MAPK pathway by increasing ROS [11]. Another study suggested that H2O2 increased ERK1/2 and AKT in HUVECs and concurrently reduced HDAC6. Subsequent treatment with the Erk1/2 inhibitor U0126 caused an increase in HDAC6, indicating that H_2_O_2_ reduces the amount of HDAC6 by stimulating ROS in a manner dependent on the Akt and ERK1/2 signaling pathways [19].

#### AKT pathway

AKT widely participates in various processes in cells and is an important physiologic pathway. This pathway has been shown to be associated with oxidative stress and HDAC6. A study suggested that the total amount of AKT and phosphorylation was increased in a mouse renal injury model, and it was inhibited by tubastatin A, revealing that HDAC6 inhibition reduces the phosphorylation of AKT [97]. GSK-3β phosphorylates Ser-22 of HDAC6 [127]. When exposed to extracts of tobacco smoke, cultured lung tissue and mouse lung tissue showed lower phosphorylation of Akt Ser473 and GSK-3β Ser9 and elevated phosphorylation of HDAC6 Ser22 in vitro; these changes were reversed by the ROS inhibitor NAC. Moreover, tobacco smoke reduced the level of ac-tubulin, suggesting that Akt inactivation and subsequent GSK-3β activation were caused by oxidative stress and that activated GSK-3β activated HDAC6 by phosphorylating Ser-22, ultimately reducing ac-tubulin and the assembly of microtubules to injure the lung endothelium [128].

**Fig. 2:**
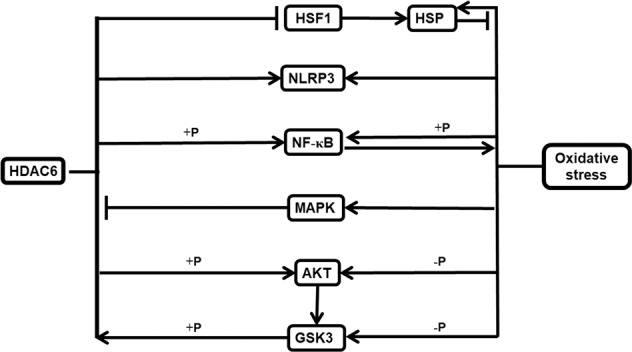
Other molecules that indirectly interact with HDAC6 and affect oxidative stress, but their roles remain to be validated. The meaning of the symbols in the figure is shown in Fig. 1

The actions of the molecules discussed in Section 3.7 are summarized in Fig. 2.

## HDAC6 Inhibition Increases Oxidative Stress in Cancer Cells

Inhibiting HDAC6 reduces the level of oxidative stress in normal tissues and cells, but in some cancer cells, inhibiting HDAC6 increases reactive oxygen species levels. This property has been used in clinical trials to treat cancers. Elevated ROS levels are a major weapon to kill cancer cells.

In melanoma-derived A375.S2 cells, the depletion of HDAC6 triggers the production of ROS and damages the mitochondrial membrane potential. HDAC6 inhibition elevates the level of Bax, which activates caspase‐3/caspase‐9 and enables the release of cytochrome C from mitochondria to the cytoplasm, leading to apoptosis in cancer cells [25]. In CAL27 human oral cavity squamous cell carcinoma cells and pharyngeal Detroit562 cells, the HDAC6 inhibitor ricolinostat increases ROS and cytotoxicity to cancer cells [27]. In rat chromephiloma PC12 cells, hypoxia and ischemia also increase ROS, and HDAC6 knockout with siRNA further increases ROS [21]. In non-Hodgkin lymphoma cells, treatment with the HDAC6 inhibitor ricolinostat induces the production of ROS and inhibits the endogenous antioxidant Trx1 [129].

In addition to cancer cells, HDAC6 inhibition induces oxidative stress injury in other types of cells. Treatment of fertilized mouse oocytes and embryos with tubastatin A increased ROS levels, suggesting that HDAC6 regulates redox homeostasis in mouse embryos [130]. In mesenchymal stem cells (MSCs), HDAC6 knockout promotes the acetylation of lysine (K) 120 in p53, significantly damaging mitochondrial respiration and increasing ROS [81].

## Conclusion

In summary, there are close links between HDAC6 and oxidative stress. HDAC6 impacts various physiologic activities in cells, and oxidative stress plays a key role in multiple pathological processes. Therapeutic modalities to modulate oxidative stress via HDAC6 show a bright future.

The specific modulatory approaches, however, present opposing results, as revealed in different studies. Currently, there are no clues about how the results were generated. According to observations in cancer cells, HDAC6 increases oxidative stress, and the cells are thereby injured. The reason may involve endogenous oxidative stress in cancer cells resulting from disturbances in peroxidase activity in cancer cells [62, 131]. In addition to cancer cells, oxidative stress in stem cells and fertilized oocytes and the role of HDAC6 inhibition need further investigation.

The inconsistent data from different studies may also result from different methods to block HDAC6. Tubastatin A, for example, targets only the DD2 domain to inhibit HDAC6, while siRNA is directed against all domains [119]. Relative to the effect on enzymatic and nonenzymatic functions by gene knockout, pharmacologic inhibition only affects enzymatic activity but not ubiquitin binding [132].

Problems with selectivity are associated with pharmacologic inhibition. Although only selective inhibitors of HDAC6 have been used, other types of HDACs may be affected, and the selectivity of different inhibitors may not be the same; these factors may exert different effects on different cells, animals, and models [17]. The study of HDAC6 should therefore use models similar to human pathologic systems to gain better use in clinical practice.
